# Predicting the Damaging Potential of Uncharacterized *KCNQ1* and *KCNE1* Variants

**DOI:** 10.3390/ijms26146561

**Published:** 2025-07-08

**Authors:** Svetlana I. Tarnovskaya, Boris S. Zhorov

**Affiliations:** 1 Sechenov Institute of Evolutionary Physiology & Biochemistry, Russian Academy of Sciences, St. Petersburg 194223, Russia; svetlanatarnovskaya@gmail.com; 2 Department of Biochemistry and Biomedical Sciences, McMaster University, Hamilton, ON L8S 4K1, Canada

**Keywords:** sequence analysis, variant annotation, protein structure, disease informatics, paralogue, missense variants

## Abstract

Voltage-gated potassium channels Kv7.1, encoded by the gene *KCNQ1*, play critical roles in various physiological processes. In cardiomyocytes, the complex Kv7.1-*KCNE1* mediates the slow component of the delayed rectifier potassium current that is essential for the action potential repolarization. Over 1000 *KCNQ1* missense variants, many of which are associated with long QT syndrome, are reported in ClinVar and other databases. However, over 600 variants are of uncertain clinical significance (VUS), have conflicting interpretations of pathogenicity, or lack germline information. Computational prediction of the damaging potential of such variants is important for the diagnostics and treatment of cardiac disease. Here, we collected 1750 benign and pathogenic missense variants of Kv channels from databases ClinVar, Humsavar, and Ensembl Variation and tested 26 bioinformatics tools in their ability to identify known pathogenic or likely pathogenic (P/LP) variants. The best-performing tool, AlphaMissense, predicted the pathogenicity of 195 VUSs in Kv7.1. Among these, 79 variants of 66 wildtype residues (WTRs) are also reported as P/LP variants in sequentially matching positions of at least one hKv7.1 paralogue. In available cryoEM structures of Kv7.1 with activated and deactivated voltage-sensing domains, 52 WTRs form intersegmental contacts with WTRs of ClinVar-listed variants, including 21 WTRs with P/LP variants. ClinPred and paralogue annotation methods consistently predicted that 21 WTRs of *KCNE1* have 34 VUSs with damaging potential. Among these, 8 WTRs are contacting 23 Kv7.1 WTRs with 13 ClinVar-listed variants in the AlphaFold3 model. Analysis of intersegmental contacts in CryoEM and AlphaFold3 structures suggests atomic mechanisms of dysfunction for some VUSs.

## 1. Introduction

Voltage-gated potassium channels Kv7.1, encoded by the gene *KCNQ1*, play important roles in the physiology and pathophysiology of the heart [1,2]. Homotetrameric Kv7.1 associated with different beta subunits, which are encoded by *KCNE* genes, are expressed in various organs including the colon, kidney, stomach, inner ear, and testis; for reviews, see [3,4]. In the heart, hKv7.1 channels mediate the slow-delayed rectifier potassium current I_Ks_, which is essential for the normal cardiac rhythm [5]. Mutations in the *KCNQ1* gene are associated with several heart diseases [3], including long QT syndrome [6,7], short QT syndrome [8,9], atrial fibrillation [10,11], and sudden cardiac death [12,13]. Recently, Kv7.1 was found to mediate sex-dependent cold sensation [14].

Each Kv7.1 subunit has six transmembrane (TM) segments. Segments S1 to S4 form the voltage-sensing domain (VSD). Segments S5 and S6, with a membrane-reentrant P-loop between them, contribute a quarter to the pore domain. The C-terminal domain, which is critical for the channel tetramerization, binds calmodulin, which regulates gating [15]. *KCNE1* is the primary accessory subunit for the cardiac channel Kv7.1 [16]. Kv7.1-mediated slow potassium current I_Ks_ plays a relatively minor role under normal heart conditions, but becomes crucial under strong adrenergic stimulation, such as during stress or exercise [17]. Other voltage-gated potassium channels involved in repolarization include Kv1.4, Kv1.5, Kv1.11, Kv4.2, and Kv4.3; see ref. [18] for a review. Thousands of disease-associated mutations in potassium channels are reported in ClinVar [19] and other databases. Here, we focus on missense disease mutations in *KCNQ1* and *KCNE1*.

As of 6 March 2025, the ClinVar database lists about 1000 disease-associated missense variants of *KCNQ1*. Among these, 519 variants are of unknown clinical significance (VUSs), 131 variants are reported with conflicting interpretations of pathogenicity (CIP), and germline classification is not provided (NP) for 70 variants. The most common diseases associated with Kv7.1 variants are long QT syndrome [20] and short QT interval syndrome [9]. The *KCNE1* β subunit has only 103 residues, but as many as 624 missense variants are reported in ClinVar. Among these, 100 variants are VUSs, 21 variants are CIPs, and 24 are NP variants. Artificial neuronal networks have been used to predict some biophysical characteristics of *KCNE1* variants [21]. Functional studies, including high-throughput electrophysiology, were employed to reclassify many Kv7.1 VUSs as P/LP variants [22] and reveal molecular mechanisms underlying pathogenicity [23]. The large number of yet uncharacterized VUSs motivates the application of computational methods to predict their damaging potential.

The American College of Medical Genetics and Genomics and the Association for Molecular Pathology recommend using in silico predictive algorithms for the interpretation of variants [24]. Many computational tools based on different principles have been developed to predict the likelihood that a mutation would damage the protein function [25,26]. The performance of in silico tools ranges between 60 and 80% and may depend on the disease phenotype [27] and the protein type [28]. For instance, MetaLR, MetaSVM, and MCap have shown top performance in predicting pathogenicity for variants associated with cardiovascular abnormalities [25]. However, some methods yielded many false-positive and false-negative predictions of pathogenicity in individual protein families [25,29]. Thus, MetaSVM predicted a pathogenic effect for 75% of benign variants of the cardiac sodium channel Nav1.5 [30]. Selecting a tool with a high success rate of correct predictions for specific protein families and adjusting the pathogenicity threshold can improve predictions.

Previously, we used various bioinformatics tools combined with the paralogue annotation method [31] to predict the damaging potential for numerous VUSs of the cardiac sodium channel Nav1.5 [30], calcium channel Cav1.2 [32], and the TRPM4 channel [33]. The paralogue annotation method employs multiple sequence alignment of functionally and structurally related proteins, focusing on residues in sequentially matching positions where a disease mutation is known for at least one family member. A VUS in the matching position of the channel under investigation is then predicted as a likely damaging (LD) variant.

In this study, we collected 1750 benign and pathogenic missense variants of Kv channels from several databases and tested 26 bioinformatics tools in identifying damaging variants. The best-performing tool, AlphaMissense, in combination with the paralogues annotation method, predicted damaging potential for 79 VUSs. We further used another tool, ClinPred, in combination with paralogue annotations to predict the damaging potential for 34 VUSs of 21 WTRs in *KCNE1*. In available cryoEM structures of Kv7.1 with activated and deactivated VSDs and in the AlphaFold model of *KCNE1*-bound Kv7.1, 52 WTRs of likely damaging VUSs form intersegmental contacts with ClinVar-listed WTRs, including 21 WTRs with P/LP variants. Analysis of state-dependent contacts revealed in 3D-aligned structures suggests an atomic mechanism of dysfunction for some VUSs.

## 2. Results and Discussions

### 2.1. Universal Residue Labels of Kv7.1 and Its Paralogues

In this study, we show UniProt residue numbers of Kv7.1 and its paralogues, as well as PLIC labels, which are universal for P-loop ion channels [34]. A PLIC label refers to the segment, the channel subunit (when necessary), and residue position relative to the reference residue in the segment, which is most conserved in the multiple sequence alignment of P-loop channels. The reference residues have numbers 550 in TM helices or 850 in P-loops. Subunits are designated “A”, “B”, “C”, and “D”. When viewed from the extracellular side, subunits A to D are arranged clockwise [35]. PLIC labels facilitate recognition of residue locations in different P-loop channels. Several 3D-aligned structures of Kv7.1 and Kv7.2 channels with PLIC labels are available in the database https://plic3da.com. Supplemental file PLIC_Uniprot_Kv.xlsx provides relations between PLIC labels and UniProt numbers for potassium channels mentioned in this study.

### 2.2. Composing a Broad Dataset of Missense Variants for Channel hKv7.1 and Its Paralogues

According to the Ensemble database, there are 31 paralogues of *KCNQ1* (ENSG00000053918), but only 14 of them have P/LP variants that are used in the paralogue annotation method. For *KCNQ1* and its 14 paralogues (Table 1), we collected a total of 5632 missense variants from the gnomAD, ClinVar, Ensembl, and Humsavar databases (Table S1). These include 1059 P/LP variants, 691 common neutral variants (with AF > 0.00001), and 3882 uncharacterized variants or VUSs. We refer to this dataset as the “broad dataset.” The largest number of P/LP variants (394) was identified in *KCNQ2*. For the hKv7.1 channel, we found 299 P/LP variants, 43 benign variants (with AF > 0.00001), and 519 VUSs (Table 1 and Table S1).

### 2.3. Distribution of Missense Variants in Topological Regions of hKv7.1

Many pathogenic variants are localized in the C-terminal region and P-loop of the channel. Most of the P/LP variants are associated with the long QT syndrome 1 (LQT1).

### 2.4. Comparing Performance of Bioinformatics Tools

**Figure 1:**
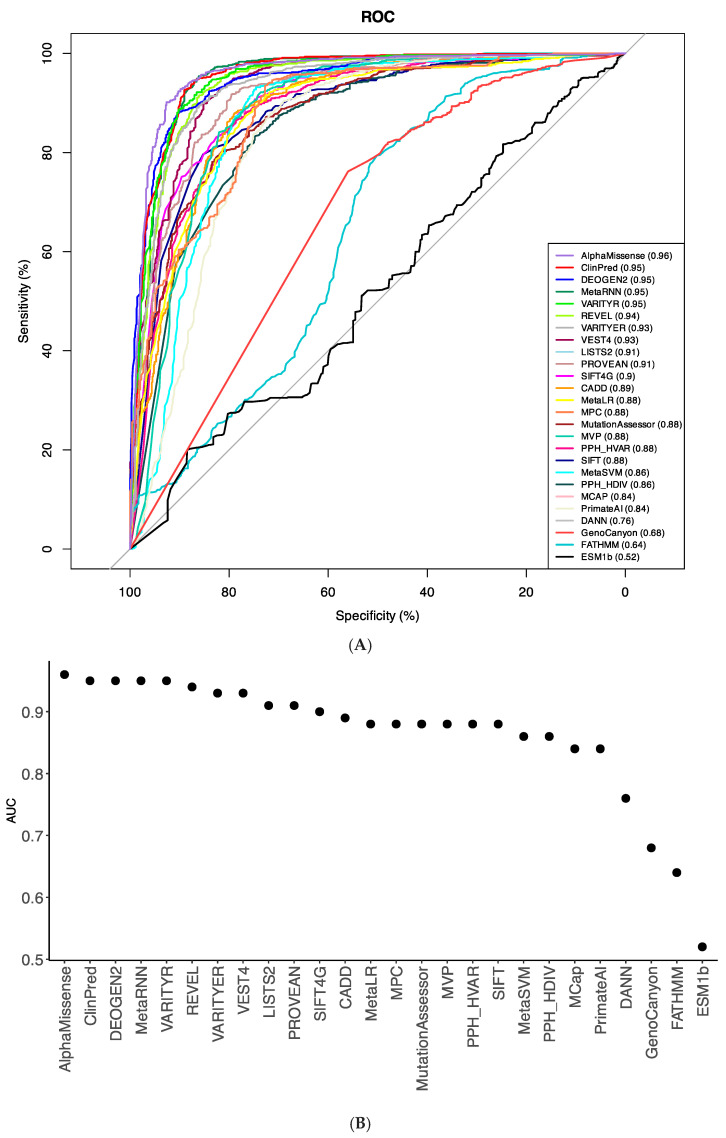
(**A**) ROC curves for prediction algorithms on the broad dataset illustrate the performance of quantitative predictions. The larger the area under the ROC curve (AUC), the better the algorithm’s performance. (**B**) AUC values arranged to decrease from the best-performing algorithm (AlphaMissense) to the worst-performing algorithm (ESMB1b).

We have chosen only those tools that predicted pathogenicity for >70% variants in our dataset. Thus, we excluded from our analysis algorithms EVE, MutPred, and MutationTaster. To compare the performance of 26 prediction tools (Table 2), we compiled a test set with 691 true-positive (TP) and 1059 true-negative (TN) observations obtained from our broad dataset (Table S1). Pre-computed algorithm scores were retrieved from the database dbNSFP v4.5. For each tool, we determined the optimal pathogenicity threshold and calculated sensitivity, specificity, and accuracy (Table 2). We used the area under the Receiver Operating Characteristic (ROC) curve as the performance measure (Figure 1A).

AlphaMissense and ClinPred demonstrated the best performance (AUC = 0.96) in the broad dataset (Figure 1), followed by VARITYR (AUC = 0.95), MetaRNN (AUC = 0.95), and DEOGEN2 (AUC = 0.95). AlphaMissense correctly classified 96% of the P/LP variants as pathogenic variants and 85% of the common neutral variants as tolerated variants (Table 2).

DEOGEN2, MetaRNN, and VARITYR also have high predictive parameters with AUC = 0.95. The lowest accuracy across all methods was found for ESM1b (AUC = 0.52), FATHMM (0.64), and GenoCanyon (0.68). The results indicate that AlphaMissense and ClinPred are the best-performing pathogenicity predictors for variants in the Kv family.

### 2.5. Paralogue Annotation of Kv7.1 Variants

Using multiple sequence alignments of hKv7.1 and its paralogues, we mapped each residue with a known P/LP variant from a paralogue protein onto the corresponding amino acid position of hKv7.1. A total of 555 known P/LP variants in paralogues were mapped to 195 amino acid positions in the hKv7.1 channel (Table S2). In these positions, we found 410 variants of hKv7.1, including 174 VUSs, 226 P/LP variants, and 10 common neutral variants. In some cases, multiple variants were mapped to the same sequence position. Forty-three P/LP variants of paralogues were mapped to the P-loop region of hKv7.1, forty-seven P/LP paralogue variants to the C-terminal region, and twenty-four P/LP variants to the S4 segment (Table S2).

### 2.6. AlphaMissense and Paralogue Annotations Consensually Predicted Damaging Potential for 79 VUSs

As many as 519 variants of Kv7.1 in our dataset are currently classified as VUSs (Table 1). We used the best-performing tool, AlphaMissense, to predict the damaging potential of these VUSs. AlphaMissense identified 195 VUSs with a pathogenicity threshold > 0.5 as P/LP variants. Among these, we further selected those variants that are annotated as P/LP in at least one of the fourteen paralogues of hKv7.1 (Table 1) with a conservation score across paralogues Cs > 0.3. Both methods consensually predicted damaging potential for 79 VUSs (Table 3).

Alfred George and co-authors [22] functionally assessed 78 Kv7.1 variants using a repurposed automated electrophysiology platform originally designed for drug discovery. The authors reclassified over 65% of the examined VUSs and found strong concordance with conventional electrophysiology results. Among the 79 likely damaging VUSs predicted in our study (Table 3), 6 were functionally characterized [22]; 5 of these exhibited a strong reduction in current density, and 1 showed a moderate decrease (Table 3). These experimental data support the predictive power of our approach. In the next section, we describe intersegmental contacts of WTRs in likely damaging VUSs, which may help guide their selection for functional studies.

### 2.7. Intersegmental Contacts Involving WTRs with Likely Damaging VUSs

**Figure 2:**
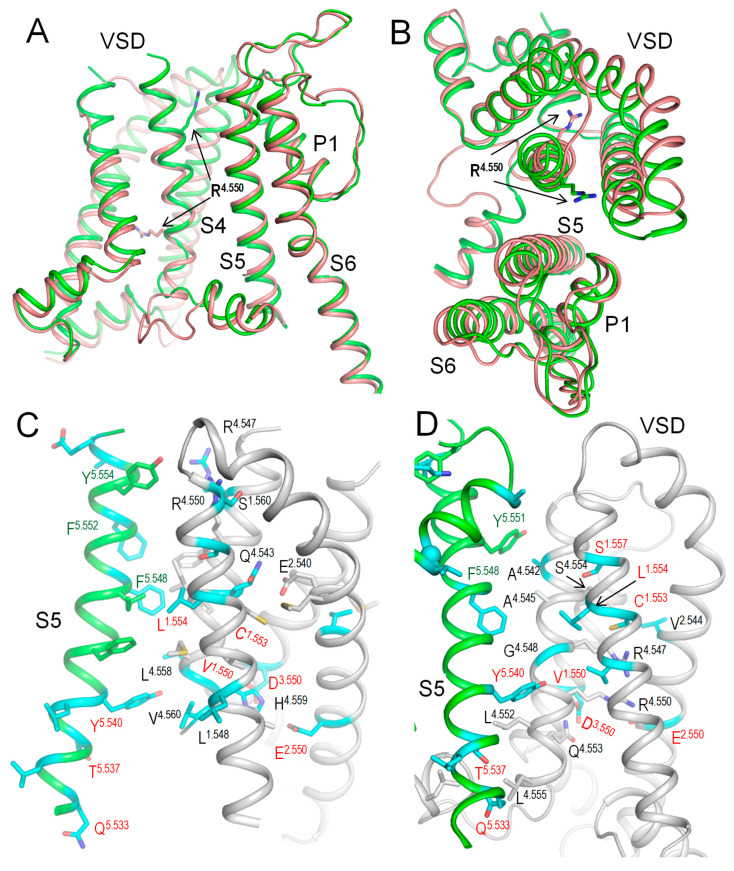
CryoEM structures of hKv7.1. (**A**,**B**) Side (**A**) and extracellular (**B**) views of the interface between a VSD and a quarter of PD in cryoEM structures with activated (green, 8sik) and deactivated (brown, 8sin) VSDs. Note a significant downshift of R^4.550^ (**A**) and its anticlockwise rotation (**B**) upon VSD deactivation. (**C**,**D**) Intersegmental contacts of WTRs with likely damaging VUSs (cyan carbons) in the cryoEM structures with activated (**C**) and deactivated (**D**) VSDs. Specific contacts are listed in Table 4, Table 5, Table 6 and Table 7. Red labels indicate residues that contact WTRs with known P/LP variants (Table 4).

**Figure 3:**
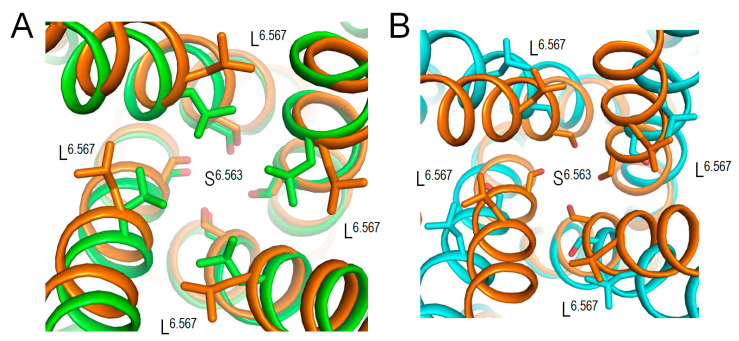
Cytoplasmic views of the Kv7.1 pore lumen in 3D-aligned cryoEM structures. (**A**) In structures with activated (8sik, green) and deactivated (8sin, brown) VSDs, the bundle of S6 helices is rather conserved, and the pore-facing serines S^6.563^ apparently block the lumen, suggesting the closed channel [36]. The pore constriction at the level of leucines L^6.576^ in 8sik is narrower than in 8sin, but this difference is mainly due to rotations of the leucine side chains. (**B**) In the structure with *KCNE3*-bound Kv7.1 and down VSDs (6v01, cyan), the bundle of S6 helices is much wider than in the Kv7.1 structure with deactivated VSD and without *KCNE* (8sin, brown). In the former structure, neither S^6.563^ nor L^6.567^ apparently blocks the pore, in agreement with the notion that *KCNE3* binding creates constitutively open Kv7.1.

The above bioinformatics approaches strongly suggest the damaging potential of 79 VUSs in Kv7.1, but the molecular mechanisms of the variants’ dysfunction are unclear. Analysis of intersegmental contacts and their state dependency may suggest mechanisms of stabilization or destabilization of specific channel states that underlie the molecular mechanisms. We this goal in mind, we 3D-aligned cryoEM structures of Kv7.1 with VSDs in the activated (8sik) and deactivated (8sin) states [36], as described in the Methods. Upon a VSD deactivation, helix S4 underwent a substantial downshift (Figure 2A) and rotation (Figure 2B), while rearrangements of helices S5 and S6 were rather small. In both 8sik and 8sin structures, the pore at the level of serines S^6.563^ is narrow (Figure 3A), implying a closed channel [36]. Surprisingly, leucines L^6.567^ in the 8sik structure form a much tighter constriction than in 8sin (Figure 3A). However, CA atoms of leucines L^6.567^ in both structures are close to each other, and respective CA-CB bonds are collinear (Figure 3A), indicating that the tighter constriction in 8sik is due to the sidechain rotations rather than rearrangement of the S6 bundle.

In epithelial cells, *KCNE3* associates with *KCNQ1* to form apparently voltage-independent channels. By using voltage clamp fluorimetry, Larsson and co-authors [37] found that *KCNE3* shifts the voltage dependence of S4 movement to extreme hyperpolarized potentials, making the channel constitutively open. These results suggest that *KCNE3* primarily affects the voltage-sensing domain and only indirectly affects the gate. In a later study, Kasuya and Nakajo used the *KCNQ1–KCNE3*–calmodulin complex structure to examine amino acid residues in *KCNE3* and the S1 segment of the *KCNQ1* [38]. By changing the side-chain bulkiness of these interacting residues, the authors found that the distance between the S1 segment and *KCNE3* is optimized to achieve constitutive activity, suggesting that the tight binding of the S1 segment and *KCNE3* is crucial for controlling the VSDs.

While VSDs in the cryoEM structure (6v01) of *KCNE3*-bound Kv7.1 are deactivated [6], the S6 bundle is much wider than that in 8sin (Figure 3B), indicating that *KCNE3* binding is sufficient to widen the pore even when VSDs are deactivated. Thus, although cryoEM structures of Kv7.1 with activated VSDs and open pore domain are lacking, available cryoEM structures of Kv7.1 allow analyzing intersegmental contacts of WTRs of likely damaging VUSs and, to some extent, their state-dependency.

**Figure 4:**
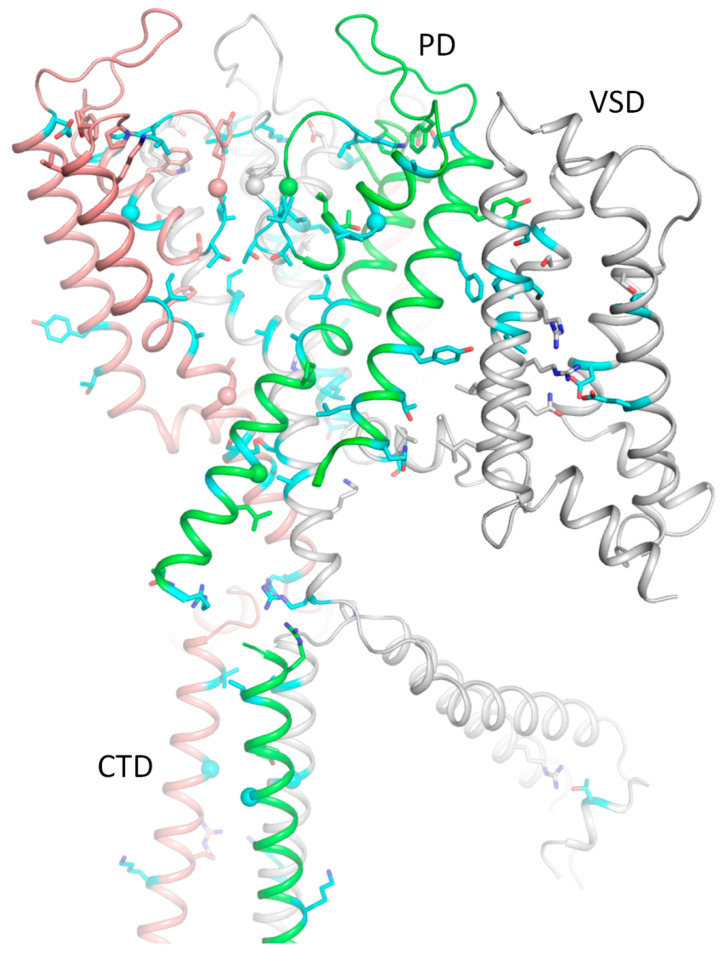
CryoEM structure of hKv7.1 with VSDs in the down state (PDB ID: 8sin). Subunit C and some other parts of the channel are removed for clarity. Subunits A, B, and D are gray, green, and brown, respectively. WTRs involved in intersegmental contacts (Table 4, Table 5, Table 6 and Table 7) are shown as sticks (CA atoms of glycines as spheres). WTRs of likely damaging VUSs (Table 3) are shown with cyan carbons, and their contact WTRs with ClinVar-listed variants are shown with carbons colored as respective ribbons. Close-up views of specific contacts are given in Figure 5 and Figure 6.

**Figure 5:**
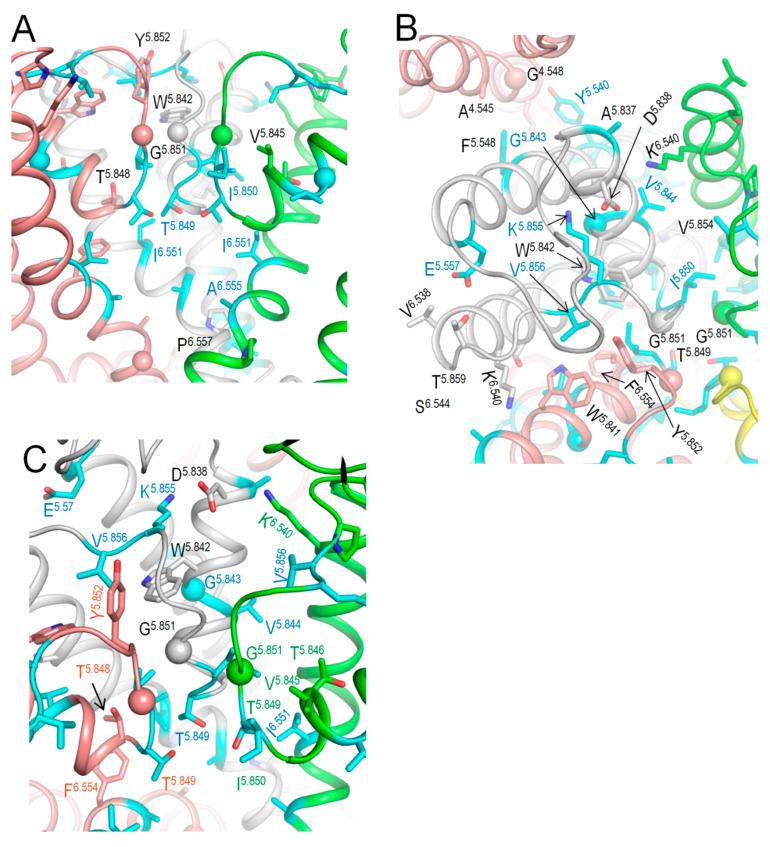
Close-up views of contacts in the extracellular part of Kv7.1 from Figure 4. WTRs involved in intersegmental contacts (Table 4) are shown as sticks, and CA atoms of glycines as spheres. WTRs of likely damaging VUSs (Table 3) are shown with cyan carbons. Their WTR contacts with ClinVar-listed variants are shown with carbons colored as respective ribbons. (**A**) View from the pore at the selectivity filter region. (**B**) Extracellular view at contacts involving P-loops. (**C**) View from the pore on contacts involving the P-loop.

**Figure 6:**
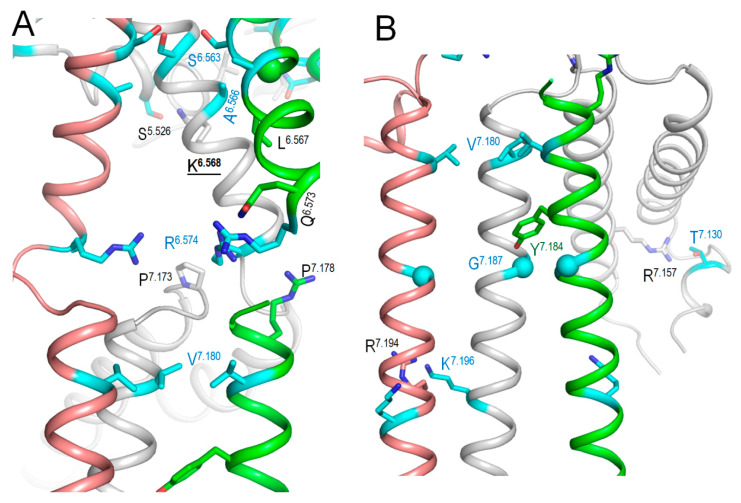
Close-up views of contacts in the cytoplasmic part of Kv7.1 from Figure 4. (**A**) View at the border between transmembrane and cytoplasmic domains. (**B**) View at the lower part of the cytoplasmic domain. WTRs with likely damaging VUSs are shown with cyan carbons.

We visualized intersegmental contacts in VSDs involving WTRs of likely damaging VUSs and WTRs of other ClinVar-reported variants in structures with deactivated (Figure 2C) and activated (Figure 2D) VSDs. Since the pore domain and especially its extracellular half are more 3D-conserved than VSDs, we analyzed intersegmental contacts in the pore domain in the structure with deactivated VSDs (Figure 4, Figure 5 and Figure 6), and then in the model with the *KCNE1*-bound channel. These contacts are listed in Table 4, Table 5, Table 6 and Table 7.

The vast majority of Kv7.1 P/LP variants, which are reported in ClinVar, as well as likely damaging VUSs (Table 3), are associated with long QT syndrome, indicating that respective mutations destabilize the open channel, decrease the I_Ks_ current, and thus prolong the action potential. In the case of the cardiac channel Nav1.5, for many intersegmental state-dependent contacts involving WTRs with ClinVar-reported variants, substitution of either contact partner causes the channel dysfunction associated with the same syndrome [39]. By analogy, we propose that if two WTRs of Kv7.1 form an intersegmental contact with one partner having a ClinVar-reported P/LP variant and another partner is a likely damaging VUS, it strongly increases the reliability of our prediction on the likely damaging VUS.

**Table 4:** Likely damaging (LD) VUSs whose WTRs contact WTRs of ClinVar-reported P/LP variants ^a^.

LD VUS	P/LP	State ^b^			LD VUS	P/LP	State	
V^133/1.550^A	R^231/4.550^L		↓		L^262/5.535^V	P^343/6.557^A		↓
	Q^234/4.553^P	↑		o		P^343/6.557^A/S	↑	
C^136/1.553^F	S^225/4.544^L/W		↓		T^264/5.537^S	L^233/4.552^M		↓
	L^156/2.536^P	↑		o		L^251/5.524^Q/P	↑	
	Q^234/4.553^P	↑				G^269/5.542^R/S/V/D	↑	
L^137/1.554^P	S^225/4.544^L/W		↓		Y^267/5.540^F	G^229/4.548^D		↓
	G^229/4.548^D		↓			L^233/4.552^M		↓
	Q^234/4.553^P	↑			E^284/5.557^G	T^322/5.859^P/A/R		↓
	I^235/4.554^N	↑				P^320/5.857^S		↓
	I^274/5.547^D	↑				G^325/6.539^W		
	R^231/4.550^S/C/L/H			o	G^306/5.843^E	L^273/5.546^I/V/P/R/F		↓
	Q^234/4.553^P			o	V^307/5.844^M/L/E	S^330/\6.544^Y		↓
	I^235/4.554^L/N			o	T^312/5.849^S	T^312/5.849^I/S		↓
S^140/1.557^R	S^225/4.544^L/W		↓		I^313/5.850^F	G^314/5.851^R/D/S		↓
	L^156/2.536^P	↑				T^312/5.849^S/I		↓
	R^231/4.550^S/C/L/H	↑		o		T^309/5.846^I		↓
	Q^234/4.553^P	↑		o	V^319/5.856^M	Y^315/5.852^S		↓
	L^156/2.536^P			o		W^304/5.841^R/L/S		↓
S^143/1.560^F	R^231/4.550^S/C/L/H			o		W^304/5.841^G		↓
V^164/2.544^A	S^209/3.557^P			o		Y^315/5.852^D		↓
E^170/2.550^G	R^231/4.550^S/H/C/L		↓		I^337/6.551^M	F^340/6.554^L		↓
D^202/3.550^V	Q^234/4.553^P		↓		F^339/6.553^V	L^251/5.524^Q		↓
	R^231/4.550^S/C/L/H		↓			L^251/5.524^P		↓
	R^234/4.562^S/C/L/H/P	↑			A^341/6.555^T	A^344/6.558^E/V		↓
	R^243/4.562^S/C/L/H/P			o	S^349/6.563^A/L	G^345/6.559^R/V/E		↓
Q^260/A5.533^H	L^251/D5.524^Q/P		↓		R^360/6.574^K/T	R^539/7.178^W/Q		↓
L^262/5.535^V	P^343/6.557^A/S			o	K^557/7.196^R	R^555/7.194^S/C		↓

^a^ ClinVar of 4 March 2025; ^b^ ↓, contacts in the hKv7.1 cryoEM structure with “down” VSDs (PDB ID: 8sin) [36]; ↑, contacts in the hKv7.1 cryoEM structure with “up” VSDs (PDB ID: 8sik) [36]; o, Contacts in hKv7.1-*KCNE3*-PIP3 cryoEM structure with open PD and “down” VSDs (PD ID: 6v01) [40].

**Table 5:** Likely damaging (LD) VUSs whose WTRs form intersegmental contacts with WTRs of other ClinVar-reported VUSs ^a^.

LD VUS	Contact ^b^		LD VUS	Contact ^b^	
V^133/A1.550^I/A	R^228/A4.547^W	VUS → LD	K^318/A5.855^N	D^301/A5.838^Y	VUS
	G^229/A4.548^S/V	VUS	S^349/A6.563^A/L	S^349/B,D6.563^A/L	VUS → LD
	Y^267/B5.540^F	VUS → LD		A^352/B,D6.566^P/D	VUS → LD
C^136/A1.553^F	R^228/A4.547^W	VUS→ LD	A^352/A6.566^P/D	L^353/B6.567^P	VUS
L^137/A1.554^P	G^229/A4.548^S/V	VUS		S^349/B6.563^A/L	VUS → LD
	R^228/A4.547^W	VUS → LD		G^350/B6.564^R	VUS
D^202/A3.550^V	Q^234/A4.553^L/R	VUS → LD	R^360/A6.574^K/T	P^535/A7.174^T	VUS
S^253/A5.526^A	K^354/A6.568^R	VUS → LD	T^391/A7.030^P	R^518/A7.157^Q/P	VUS
Y^267/A5.540^F	G^229/D4.548^S/V	VUS	V^541/A7.180^I	V^541/B,D7.180^I	VUS → LD
E^284/A5.557^G	V^324/6.538^L/I/F	VUS		Y^545/B7.184^F	VUS
A^300/A5.837^S/G	K^326/B6.540^E	VUS	G^548/A7.187^D	Y^545/B7.184^F	VUS
T^312/A5.849^S	I^313/5.850^F	VUS → LD			
	I^337/A6.551^M	VUS → LD			

^a^ ClinVar Data on 4 March 2025; ^b^ In the cryoEM structure of hKv7.1 (PDB ID: 8sin) with voltage sensor in the down state [36].

**Table 6:** Intersegmental contacts ^a^ between WTRs of likely damaging (LD) VUSs and WTRs of CIP or NP variants ^b^.

LD VUS	Contact		LD VUS	Contact	
V^164/A2.544^A	S^209/A3.557^P	NP	I^313/A5.850^F	G^314/B5.851^C/A	NP
A^223/A4.542^T	Y^278/B5.551^H	NP		T^309/B5.846^S/R	NP
Q^260/A5.533^H	L^236/D4.555^R/P	CIP		V^308/B5.845^D	NP
T^264/A5.537^S	L^236/D4.555^R/P	CIP	K^318/A5.855^N	D^301/A5.838^V	CIP
	L^233/D4.552^P	CIP	I^337/A6.551^M	T^311/D5.848^A	NP
Y^267/A5.540^F	L^233/4.552^P	CIP		T^311/D5.848^I	CIP
F^275/A5.548^L	A^226/D4.545^V	CIP	A^341/A6.555^T	A^344/D6.558^T/G	CIP
E^284/A5.557^G	T^322/5.859^K	CIP	S^349/A6.563^A/L	S^349/B,D6.563^P	CIP
	F^296/5.611^S	CIP	A^352/A6.566^P/D	S^349/B6.563^P	CIP
T^312/A5.849^S	I^337/A6.551^F	CIP		G^350/B6.564^V	CIP
			T^391/A7.030^P	R^518/A7.157^G	CIP

^a^ In the cryoEM structure of hKv7.1 (PDB ID: 8sin) with the voltage sensor in the down state [36]; ^b^ ClinVar Data of 4 March 2025.

**Table 7:** CryoEM structure ^a^ with activated VSDs: intersegmental contacts of WTRs in VSD and S5 ^b^.

LD VUS	Contact	
C^136/A1.553^F	M^159/2539^L	VUS
S^140/A1.557^R	Q^260/A5.533^H	LD VUS
V^164/A2.544^A	M^210/3558^T/I	VUS
E^170/A2.550^G	H^240/4.559^Q	LD VUS
	V^129/1546^I/G/A	VUS
D^202/A3.550^V	H^240/4.559^Q	LD VUS
L^239/4.558^V	Y^267/A5.540^F	LD VUS
	F^275/A5.548^S/L	LD VUS
H^240/4.559^Q/R	E^170/A2.550^G	LD VUS
	D^202/A3.550^H	LD VUS
V^241/4.560^I	Y^267/5.540^F	VUS
Y^267/A5.540^F	V^241/4.560^I	LD VUS
	L^239/4.558^V	LD VUS
F^275/A5.548^L	L^239/4.558^V	LD VUS

^a^ In the cryoEM structure of hKv7.1 (PDB ID: 8sik) with the voltage sensor in the up state [36]; ^b^ ClinVar Data of 4 March 2025.

Such contacts with known P/LP residues involve 31 likely damaging VUSs of 21 WTRs, including eight variants in VSD, seven variants in S5, eight variants in P-loop, and eight variants in S6 (Table 4). Contacts within a VSD and between VSD and S5 are clearly state-dependent (cf. Figure 2C,D). In particular, H-bonds R^4.550^---S^1.560^, Q^4.553^---E^2.540^_,_ salt bridges H^4.559^: E^2.550^ and H^4.559^: D^3.550^, and hydrophobic interactions L^4.558^: F^5.548^ stabilize the “up” state of S4 and thus activated state of VSD (Figure 2D). Mutations R^4.550^L, D^3.550^H, Q^4.553^P, E^2.550^G (Table 4), L^4.558^V, and F^275/A5.548^S/L (Table 7) would weaken or eliminate these contacts and destabilize the activated VSD. This would decrease the probability of the channel open state, reduce I_Ks_, and prolong the action potential in cardiomyocytes, explaining why respective variants are associated with the gain-of-function long QT syndrome. However, some of the above mutations (e.g., R^4.550^L and E^2.550^G) would also destabilize deactivated VSD (Figure 2D). Therefore, the long QT syndrome associated with these variants indicates that respective mutations destabilize the activated state of VSD more strongly than its deactivated state.

Table 4 also shows seven likely damaging VUSs in the cytoplasmic part of S6 (positions 6.553–6.574) and six likely damaging VUSs in the cytoplasmic part of S5 (positions 5.533–5.540). These residues undergo substantial movements upon activation gating, implying that respective mutations destabilize the channel open state. Importantly, WTRs of the respective variants contact WTRs with ClinVar-reported P/LP variants (Table 4). These residues are marked with red labels in Figure 4.

The second category of contacts includes 16 WTRs of 26 likely damaging VUSs and 25 partner WTRs of 35 ClinVar-reported VUSs (Table 5). Among the contact partners, 13 WTRs have 18 VUSs that we also predicted as likely damaging variants (Table 4). The fact that VUSs of both partners in such contacts were independently selected by AlphaMissense and paralogue annotation methods further justifies our predictions that respective VUSs are likely damaging, making them promising objects for future functional analyses.

The third category of contacts includes 15 WTRs of 20 likely damaging VUSs and 16 contact-partner WTRs for which 26 variants are reported in ClinVar as either CIP variants or their germline classification is not provided (Table 6). We suggest a damaging potential of the CIP variants because at least one submitter reported likely pathogenicity of the variant, and the respective WTR is contacting a WTR with a likely damaging VUS.

We found four WTRs of ClinVar-reported benign/likely benign (B/LB) variants that form intersegmental contacts with WTRs of likely damaging VUSs (Supplementary Figure S1). Among these contacts, only B/LB mutation S^1.560^F would affect the polar contact with T^2.553^ and thus the stability of the VSD state.

### 2.8. Predicting Damaging Potential for KCNE1 VUSs with High ClinPred Score and ClinVar-Reported Variants in Sequentially Matching Positions of Paralogues

*KCNE1* association with Kv7.1 is important for normal heart rhythm; for reviews, see [5,16]. As of March 2025, over 600 variants of *KCNE1* are reported in ClinVar, but only three missense variants (T^7^I, M^1^L, and G^52^R) are classified as pathogenic. Among four known *KCNE1* paralogs (*KCNE2, KCNE3, KCNE4*, and *KCNE5*), *KCNE2* has five P/LP variants, while for the other paralogues, only VUSs are reported. Therefore, the paralogues annotation method for *KCNE* subunits is less reliable than for Kv channels. Since AlphaMissense correctly predicted the damaging effect of fewer than 60% of *KCNE* variants, we used the second top-performing tool (Section 2.4), ClinPred, with the standard cutoff score (deleterious threshold) of 0.5. ClinPred and the paralogue annotations consensually predicted the damaging potential of 34 VUSs for 21 WTRs in *KCNE1* (Table 8). Among the 34 VUSs, 20 variants were explored in a recent high-throughput functional study of 68 *KCNE1* variants [41], and for most of these, the pick current decrease in the *KCNQ1-KCNE1* assembly is reported (Table 8). These experimental data confirm the reliability of our approach to predict the damaging potential of *KNCE1* variants.

### 2.9. AlphaFold 3 (AF3) Model of Kv7.1 with KCNE1

**Figure 7:**
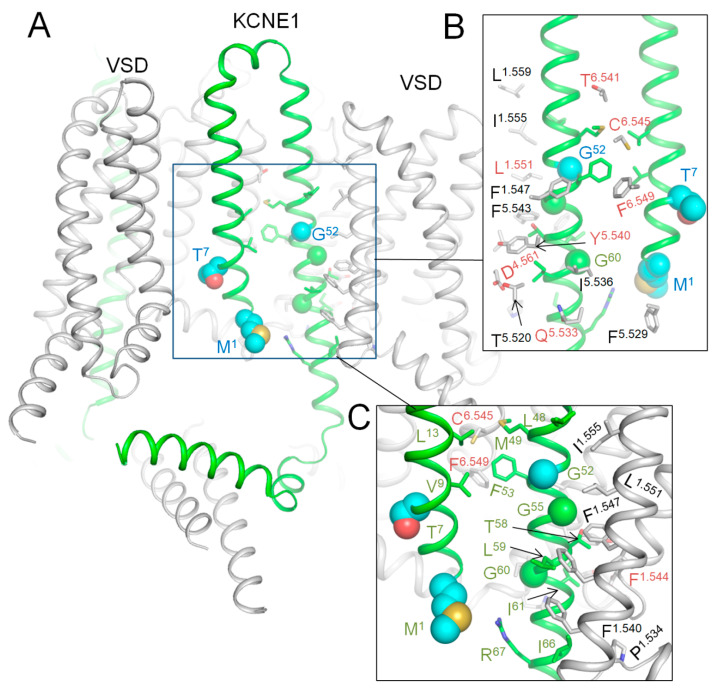
AF3 model of *KCNE1*-bound hNav7.1. (**A**) Membrane view. WTRs of ClinVar-reported *KCNE1* pathogenic missense variants are shown as spheres with cyan carbons. (**B**,**C**) Close-up views of *KCNE1* contacts with Kv7.1. Backbones of Kv7.1 are not shown for clarity in panel (**B**). *KCNE1* WTRs with likely damaging variants (Table 8) are shown with green carbons. Kv7.1 residues that contact *KCNE1* are shown with gray carbons. Residues listed in ClinVar are marked with red labels (Table 9).

In the lack of cryoEM structures of Kv7.1-*KCNE1*, we created an AF3 multimer model [42,43] of complex Kv7.1-*KCNE1* and Monte Carlo (MC) minimized its energy with rigid backbones (Figure 7). In this model, the large N-terminal portion of each *KCNE1* folded up to fill the space between two VSDs, while the smaller C-terminal part extended to the cytoplasm. WTRs of pathogenic variants M^1^L, T^7^I, and G^52^R are shown as blue spheres. Likely damaging VUSs of *KCNE1* (Table 8) are shown as sticks in close-up views of Figure 7. Among multiple Kv7.1 WTRs that form contacts with *KCNE1*, six have known P/LP variants, and several others have likely damaging VUSs (Table 3). Since these data alone are insufficient to prove our AF3 model of Kv7.1-*KCNE1*, we compared the model with the cryoEM structure of *KCNE3*-bound Kv7.1 as described in the next section.

### 2.10. CryoEM Structure of KCNE3-Bound Kv7.1

**Figure 8:**
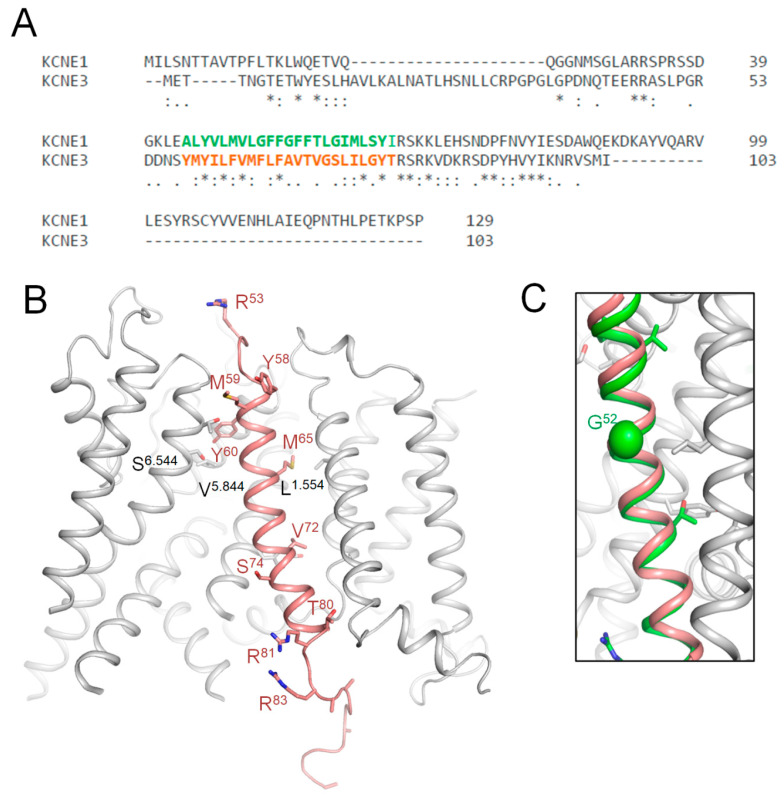
*KCNE3*-bound Kv7.1 (6v01) vs. AF3 model of *KCNE1*-bound Kv7.1. (**A**) Sequence alignment of *KCNE1* and *KCNE3*. TM helix of *KCNE3* in the cryoEM structure (Y^58^ to T^80^, orange) and the TM helix in the AF3 model (A^44^ to I^66^, green) have 6 identical and 12 similar residues. (**B**) CryoEM structure of Kv7.1 (gray) with resolved part of *KCNE3* (brown). Shown are *KCNE3* WTRs with ClinVar-reported P/LP variants (Table 8) and WTRs of their Kv7.1 contacts with likely damaging VUSs (Table 3). (**C**) TM helix of *KCNE1* in the AF3 model (green) matches the *KCNE3* helix (brown) in the cryoEM structure (6v01).

Kv7.1 co-assembles with *KCNE3* in non-excitable cells [40,44], but there is evidence that such complexes are also expressed in the human heart, especially in diseased hearts [45]. *KCNE1* and *KCNE3* sequences are very different (Figure 8A), but there is sequence similarity in the middle part where *KCNE3*, which is resolved in the cryoEM structure (6v01) of *KCNE3*-bound Kv7.1 [40], where the TM helix is bound between S1 of VSD and the extracellular parts of helices S5 and S6 (Figure 8B). In the 3D-aligned cryoEM structure of Kv7.1-*KCNE3* and the AF3 model of Kv7.1-*KCNE1*, the TM part of the *KCNE3* helix (M^60^ to T^80^) overlaps with the *KCNE1* helix from L^45^ to I^66^ (Figure 8C). It should be noted that the full-fledged cryoEM structure and the AF3 model are 3D-aligned by minimizing RMS deviations of sequentially matching CA atoms in P-loop helices P1 (see Methods) rather than sequentially matching CA atoms in the *KCNE* TM helices (Figure 8A). The perfect 3D match of the TM helices validates the position of the respective *KCNE1* TM helix in our AF3 model (Figure 7).

## 3. Methods and Materials

### 3.1. Sequence Data of Human Channels and Collection of Variants

The sequence of hKv7.1 was obtained from the UniProt database [46] (accession number P51787). Paralogues of the hKv7.1 channel were identified using Ensembl [47]. Missense mutations for Kv7.1 and its paralogues were collected from three databases: Humsavar, Ensembl Variation [48], and ClinVar [49]. Only P or LP variants were extracted from Ensembl Variation, ClinVar, and Humsavar. VUSs were also obtained from ClinVar. Common benign (neutral) variants, along with their minor allele frequencies (AF), were sourced from the population database gnomAD [50]. Variants with AF > 0.00001 that are not present in ClinVar were considered benign [51,52]. The total number of collected P/LP, VUS, and common neutral variants is given in Table 1. All variants were compiled into a comprehensive dataset (Table S1).

### 3.2. Topology of the Kv7.1 Channel

The hKv7.1 regions were defined in accordance with the UniProt entry P51787. The pore-forming α1 subunit of Kv7.1 assembles from four subunits. A large cytoplasmic domain plays critical roles in subunit assembly, interactions with regulatory proteins, and modulation of channel function [53].

### 3.3. Multiple Sequence Alignment and Paralogue Annotation

The paralogue annotation method identifies P/LP missense variants by transferring annotations across families of related proteins [31]. Previously, we applied a modified version of this method to predict likely damaging variants for channels hNav1.5 [30], hCav1.2 [32], and TRPM4 [33]. Here, we used the same approach to predict likely damaging variants for VUSs of hKv7.1.

For each paralogue channel, P/LP variants were collected. The amino acid sequences of hKv7.1 and its paralogue channels were aligned using the multiple sequence alignment program Tcoffee [54]. Proteins lacking P/LP variants were excluded from the alignment [26]. Since disease-causing mutations tend to occur at evolutionarily conserved positions, we computed position-specific conservation scores (Cs), which range from 0 (no conservation) to 1 (identical), with Cs = 0.8 indicating high conservation. These scores reflect the conservation of physicochemical properties (small, polar, hydrophobic, tiny, charged, negative, positive, aromatic, aliphatic, and proline) in the alignment [55]. Cs values were calculated using the Zvelebil method [56], as implemented in the Amino Acid Conservation Calculation Service [57]. Variants occurring at positions with Cs > 0.3 were classified as P/LP variants.

### 3.4. Sequence-Based Prediction of Likely Damaging Variants

Missense variants were annotated with scores from 29 algorithms (REVEL, VEST4, MVP, CADD, LIST.S2, VARITY_R, VARITY_ER, AlphaMissense, EVE, MPC, MVP, DANN, CenoCanyon, PrimateAI, DEOGEN2, M-CAP, MetaLR, MetaSVM, MetaRNN, FATHMM, PROVEAN, MutationAssessor, MutPred, PolyPhen2-HVAR, PolyPhen2-HDIV, SIFT, SIFT4G, LRT, MutationTaster), which were obtained from the dbNSFPv4.5 database [58]. To generate binary predictions (Damaging/Tolerated), we used thresholds determined as the optimal pathogenicity threshold from the AUC-ROC curve (Table 2).

The ‘probably damaging’ and ‘possibly damaging’ classes predicted by Polyphen were merged into a single ‘damaging’ class. For results from the MutationAssessor server, which subdivides mutants into four categories, we treated categories high (‘H’) or medium (‘M’) as ‘Damaging’, and categories low (‘L’) or neutral (‘N’) as ‘Tolerated’.

The overall prediction performance of the 29 methods was assessed by calculating sensitivity, specificity, Matthews Correlation Coefficient (MCC), and accuracy (ACC) as follows:

(1) $$Sensitivity=\frac{TP}{TP+FN};$$

(2) $$Specificity=\frac{TN}{TN+FP};$$

(3) $$MCC=\frac{TP\times TN-FP\times FN}{\sqrt{\left(TP+FP)(TP+FN)(TN+FP)(TN+FN\right)}};$$

(4) $$ACC=\frac{TP+TN}{TP+FP+TN+FN}.$$

The following abbreviations are used in these equations:

*TP* (true-positive) is the number of disease-causing variants correctly predicted to be pathogenic;
*FN* (false-negative) is the number of disease-causing variants incorrectly predicted as tolerated;
*TN* (true-negative) is the number of neutral variants correctly predicted as tolerated;
*FP* (false-positive) is the number of neutral variants incorrectly predicted as pathogenic;
*MCC* is a correlation coefficient between the observed and predicted binary classification, ranging from −1 (total disagreement between prediction and observation) to 1 (perfect prediction).

For the test dataset, we selected common neutral and P/LP variants from our comprehensive dataset (Supplementary Table S1). We also calculated the area under the ROC (Receiver Operating Characteristic) curve (AUC) using the pROC library in the R programming language. ROC curves were generated by plotting sensitivity against (1—specificity) at each threshold for each algorithm. The AUC can range from 0 (completely random) to 1 (perfectly correct prediction). The absence of a variant annotation negatively impacts prediction accuracy. Therefore, we included only those algorithms that predicted the pathogenicity of over 30% of variants in our dataset (Table S1).

### 3.5. Molecular Modeling

All computations were performed with the freely available ZMM program (www.zmmsift.ca). Energy was calculated using the AMBER force field [59] with environment- and distance-dependent dielectric function [60]. Energy was optimized with Monte Carlo (MC) minimizations [61] in the space of generalized coordinates [62]. We used the AlphaFold3 (AF3) server (https://alphafold.ebi.ac.uk) to predict multimeric complexes of hKv7.1 with four full-fledged *KCNE1* subunits. CryoEM structures of Kv7.1 with the activated and deactivated VSDs, as well as AF3 models, were imported by the ZMM program, and side chain conformations were MC-minimized with rigid backbones. MC-minimizing trajectories were terminated when the last 100th energy minimization did not improve the protein energy. All structures were 3D-aligned by minimizing the root mean square deviations of C^α^ atoms in P1 helices against a reference crystal structure of the chimeric potassium channels Kv1.2-Kv2.1 channel (PDB ID: 2R9R), the first eukaryotic P-loop potassium channels whose crystal structure was obtained with a resolution below 2.5 Å [63].

Intersegmental contacts are defined as those where two sidechains are within 5Å from each other. Such contacts were automatically identified by ZMM in MC-minimized structures. We used PyMol v099 (Schrödinger, New York, NY, USA) to visualize cryoEM structures and AF3 models of the Kv7.1 channel and its complex with *KCNE1*. Other details of computations may be found elsewhere [64].

## 4. Conclusions

In this study, we compiled a comprehensive dataset encompassing known pathogenic/likely pathogenic (P/LP) variants of the hKv7.1 channel and its 14 paralogues. Our analysis identified AlphaMissense as the top-performing bioinformatics tool for predicting (P/LP) variants in Kv channels. AlphaMissense and the paralogue annotations method consensually predicted 79 VUSs of Kv7.1 as likely damaging (LD) variants. We further predicted that 34 *KCNE1* VUSs are LD variants. Many wildtype residues (WTRs) of LD variants make state-dependent intersegmental contacts with WTRs of known P/LP variants or LD variants in cryo-EM structures or AlphaFold3 models, suggesting atomic mechanisms of the variants’ dysfunction. Respective variants of Kv7.1 and *KCNE1* are promising objects for future functional analyses.

### Study Limitations

Our computations predicted the damaging potential of many reported disease-associated mutations in *KCNQ1* and *KCNE1*, but functional studies are necessary to reclassify respective VUSs as P/LP variants. Since the cryoEM structures of Kv7.1 considered here do not provide relations between VSD activation and the pore opening, mutations with likely damaging potential within the VSD do not necessarily affect the I_Ks_ current; functional studies are necessary to explore these variants. We analyzed contacts of WTRs but not respective VUSs and suggested that mutations would modify these contacts. Despite these limitations, we hope that our study, which incorporates paralogue annotations and analysis of experimental and AF3-predicted 3D structures, provides more reliable predictions of the damaging potential of VUSs than more traditional bioinformatics approaches.

## Figures and Tables

**Table 1:** Known missense variants of hKv7.1 and its paralogues.

Gene ^a^	UniProt ID ^b^	P/LP ^c^	VUS ^d^	Common Neutral ^e^
*KCNA1*	Q09470	38	245	14
*KCNA2*	P16389	44	207	10
*KCNA5*	P22460	4	276	56
*KCNB1*	Q14721	87	212	62
*KCNC1*	P48547	11	149	11
*KCNC2*	Q96PR1	12	44	37
*KCNC3*	Q14003	10	173	82
*KCND2*	Q9NZV8	7	176	22
*KCND3*	Q9UK17	21	207	17
*KCNQ1*	P51787	299	519	43
*KCNQ2*	O43526	394	474	57
*KCNQ3*	O43525	39	458	49
*KCNQ4*	P56696	28	153	63
*KCNQ5*	Q9NR82	20	228	65
*KCNV2*	Q8TDN2	45	361	103

^a^ Genes of voltage-gated potassium ion channels, which have P/LP variants in public databases; ^b^ Accession number of a protein in the UniprotKB database; ^c^ Number of P/LP variants; ^d^ Number of VUSs; ^e^ Number of variants from the gnomAD database, which occur in a population with allele frequency > 0.00001 and are absent in the ClinVar database.

**Table 2:** Performance of variant interpretation tools.

Tool	Deleterious Threshold ^a^	Sensitivity ^b^	Specificity ^c^	MCC ^d^	ACC ^e^	AUC ^f^
AlphaMissense	>0.5	0.95	0.86	0.81	0.9	0.96
ClinPred	>0.8	0.97	0.79	0.77	0.87	0.95
DEOGEN2	>0.5	0.96	0.67	0.65	0.81	0.95
MetaRNN	>0.6	0.97	0.82	0.79	0.89	0.95
VARITYR	>0.5	0.93	0.86	0.78	0.89	0.95
REVEL	>0.45	0.99	0.56	0.59	0.76	0.94
VARITYER	>0.5	0.9	0.85	0.75	0.87	0.93
VEST4	>0.5	0.97	0.73	0.72	0.85	0.93
LISTS2	>0.85	0.97	0.54	0.55	0.73	0.91
PROVEAN	<−1.5	0.96	0.62	0.61	0.78	0.91
SIFT4G	<0.05	0.9	0.75	0.65	0.82	0.9
CADD	>3	0.93	0.68	0.63	0.8	0.89
MetaLR	>0.4	0.99	0.15	0.25	0.54	0.88
MPC	>2	0.57	0.92	0.51	0.74	0.88
MutationAssessor	>1.7	0.89	0.68	0.58	0.78	0.88
MVP	>0.75	0.99	0.43	0.49	0.68	0.88
PPH_HVAR	>0.45	0.91	0.69	0.61	0.79	0.88
SIFT	<0.0045	0.82	0.8	0.62	0.81	0.88
MetaSVM	>0	0.99	0.28	0.37	0.61	0.86
PPH_HDIV	>0.45	0.93	0.56	0.52	0.73	0.86
MCap	>0.05	1	0.19	0.31	0.57	0.84
PrimateAI	>0.6	0.98	0.46	0.5	0.7	0.84
DANN	>0.5	1	0.04	0.14	0.52	0.76
GenoCanyon	>0.7	0.89	0.35	0.29	0.62	0.68
FATHMM	<−1	0.99	0.08	0.18	0.5	0.64
ESM1b	<−3	0.69	0.35	0.05	0.51	0.52

^a^ Deleterious threshold is the custom pathogenicity threshold that divides variants into two categories: pathogenic or benign. The larger or smaller the score is than the threshold, the more likely the variant is damaging. ^b^ Sensitivity characterizes the number of P/LP variants, which were predicted as P/LP by the tool. ^c^ Specificity characterizes the number of benign variants, which were predicted as benign by the tool. ^d^ MCC, Matthews Correlation Coefficient. ^e^ ACC, Accuracy indicates the predictive accuracy of the tool. ^f^ AUC, Area under the ROC curve.

**Table 3:** Likely damaging VUSs of hKv7.1 ^a^.

PLIC Label	hKv7.1 Variant	Paralogue	ClinPred	α Missense	Current Change ^b^
1.532	E^115^D	*KCNQ2*-E^86^K	0.878	0.997	
1.545	A^128^P	*KCNQ2*-Y^98^X	0.185	0.861	
1.548	L^131^P	*KCNQ2*-L^101^H	0.995	0.958	↓↓
1.550	V^133^A	*KCNA1*-I^177^N, *KCNB1*-I^199^F, *KCNQ2*-V^103^D	0.984	0.964	↓
1.553	C^136^F	*KCNQ2*-C^106^G	1	0.954	↓↓
1.554	L^137^P	*KCNQ2*-L^107^F	0.986	0.999	
1.557	S^140^R	*KCNA1*-F^184^C	0.996	0.999	↓↓
1.560	S^143^F	*KCNB1*-N^209^K	0.997	0.953	
2.544	V^164^A	*KCNQ2*-I^134^N	0.998	0.797	
2.550	E^170^G	*KCNQ2*-E^140^A	1	0.987	
2.551	Y^171^H	*KCNV2*-Y^317^X	0.998	0.998	
2.556	W^176^S	*KCNQ2*-W^146^X	1	0.926	
2.609	G^189^A	*KCNQ2*-G^159^E/R/V	0.998	0.978	↓↓
3.549	I^201^V	*KCNA2*-I^258^N	0.944	0.507	
3.550	D^202^V	*KCNQ2*-D^172^G	1	0.998	
3.560	V^212^A	*KCNQ2*-V^182^M	0.994	0.958	
3.560	V^212^F	*KCNQ2*-V^182^M	0.997	0.917	
3.600	G^219^E	*KCNA1*-E^283^K, *KCNQ2*-G^189^D	0.989	0.817	
4.542	A^223^T	*KCNQ2*-A^193^D/V	0.994	0.772	
4.547	R^228^W	*KCNA2*-R^294^H, *KCNQ2*-R^198^W	1	0.966	
4.553	Q^234^L	*KCNA2*-R^300^S, *KCNB1*-R^303^Q, *KCNC3*-R^420^H, *KCNQ2*-Q^204^H	0.998	0.965	
4.553	Q^234^R	*KCNA2*-R^300^S, *KCNB1*-R^303^Q, *KCNC3*-R^420^H, *KCNQ2*-Q^204^H	0.97	0.992	
4.558	L^239^V	*KCNQ2*-I^209^S/T	0.829	0.86	
4.559	H^240^R	*KCNQ2*-R^210^C/H/P	0.331	0.949	
4.559	H^240^Q	*KCNQ2*-R^210^C/H/P	0.993	0.991	
5.518	G^245^R	*KCND3*-S^304^F	0.998	0.998	↓↓
5.523	L^250^P	*KCNA1*-I^314^T, *KCNB1*-S^319^F/Y	0.995	1	
5.525	G^252^R	*KCNB1*-G^321^S	1	0.996	
5.525	G^252^S	*KCNB1*-G^321^S	0.998	0.885	
5.526	S^253^A	*KCNQ2*-S^223^F/P	0.972	0.792	
5.530	I^257^S	*KCNQ2*-A^227^V	0.993	0.947	
5.533	Q^260^H	*KCNQ2*-K^230^M	0.991	0.99	
5.535	L^262^V	*KCNC3*-F^448^L	0.996	0.952	
5.536	I^263^K	*KCNB1*-G^332^V	0.997	0.997	
5.537	T^264^S	*KCNQ2*-T^234^A/P	0.992	0.895	
5.540	Y^267^F	*KCNQ2*-Y237C	0.996	0.644	
5.541	I^268^V	*KCNC2*-F^388^S	0.878	0.674	
5.548	F^275^L	*KCNQ2*-L^245^P	0.996	0.986	
5.552	F^279^C	*KCND3*-V^338^E, *KCNQ2*-L^249^P	0.999	0.788	
5.556	A^283^T	*KCNQ2*-A^253^S/T	0.996	0.504	
5.557	E^284^G	*KCNQ2*-E^254^D	1	0.972	
5.613	S^298^R	*KCNQ2*-T^263^A/I	0.997	0.977	
5.837	A^300^G	*KCNQ2*-A^265^P/T/V	0.986	0.747	
5.843	G^306^E	*KCNQ2*-G^271^D/R/S/V, *KCNQ3*-G^310^D/V	0.999	0.999	
5.844	V^307^M	*KCNB1*-T^372^N/I	0.991	0.79	
5.844	V^307^L	*KCNB1*-T^372^N/I	0.99	0.895	
5.844	V^307^E	*KCNB1*-T^372^M/I	0.993	0.989	
5.849	T^312^S	*KCNA2*-T^374^A, *KCNC2*-T^437^A, *KCNQ2*-T^277^N/P/S	0.995	0.966	
5.850	I^313^F	*KCNQ2*-I^278^F/M/T, *KCNQ3*-I^317^M/T	0.993	0.991	
5.855	K^318^N	*KCNQ2*-K^283^E	0.958	0.93	
5.856	V^319^M	*KCNQ2*-Y^284^C/D	0.995	0.616	
6.543	A^329^V	KCND3-G384S, KCNQ2-A294G/S	0.999	0.979	
6.543	A^329^T	KCND3-G384S, KCNQ2-A294G/S	0.987	0.935	
6.544	S^330^Y	*KCND3*-S^385^P	0.998	0.993	
6.545	C^331^Y	*KCNQ2*-T^296^P	0.998	0.976	
6.549	F^335^C	*KCNA1*-A^395^S	0.997	0.856	
6.551	I^337^M	*KCNA2*-V^399^M	0.962	0.775	
6.553	F^339^V	*KCNQ2*-F^304^C, *KCNQ2*-F^304^S	0.999	0.972	
6.555	A^341^T	*KCNA1*-A^401^V, *KCNB1*-A^406^V, *KCNQ2*-A^306^P/T/V/E	0.996	0.976	
6.563	S^349^A	*KCNB1*-N^414^D	0.995	0.92	
6.563	S^349^L	*KCNB1*-N^414^D	0.999	0.995	
6.566	A^352^P	*KCNB1*-S^417^P, *KCNQ2*-A^317^T, *KCNQ3*-A^356^T	0.999	0.997	
6.566	A^352^D	*KCNB1*-S^417^P, *KCNQ2*-A^317^T, *KCNQ3*-A^356^T	0.998	1	
6.569	K^354^R	*KCNQ2*-K^319^E	0.992	0.768	
6.571	Q^357^R	*KCNA1*-R^417^X, *KCNB1*-E^422^A	0.996	0.984	
6.571	Q^357^E	*KCNA1*-R^417^X, *KCNB1*-E^422^A	0.939	0.566	
6.574	R^360^T	*KCNQ2*-R^325^G, *KCNQ3*-R^364^C/H	0.999	0.999	
6.574	R^360^K	*KCNQ2*-R^325^G, *KCNQ3*-R^364^C/H	0.992	0.976	
7.013	L^374^V	*KCNQ2*-L^339^Q, *KCNQ2*-L^339^R	0.995	0.85	
7.030	T^391^P	*KCNQ2*-T^359^K	0.998	0.898	
7.165	K^526^E	*KCNQ2*-K^552^N	0.932	0.967	
7.165	K^526^Q	*KCNQ2*-K^552^N	0.838	0.717	
7.165	K^526^N	*KCNQ2*-K^552^N	0.984	0.995	
7.180	V^541^I	*KCNQ2*-V^567^D	0.903	0.814	
7.187	G^548^D	*KCNQ2*-G^574^D/S	0.999	0.999	
7.194	R^555^L	*KCNQ2*-R^581^G	0.998	0.994	
7.196	K^557^R	*KCNQ2*-K^583^N	0.982	0.63	
7.222	R^583^S	*KCNQ2*-K^606^X	0.885	0.902	
7.222	R^583^G	*KCNQ2*-K^606^X	0.79	0.606	
7.230	R^591^P	*KCNQ2*-R^622^P	0.997	0.997	

^a^ Shown are PLIC labels that are universal for P-loop channels [34] and UniProt residue numbers of specific channels. ^b^ Current density decreases strongly (↓↓) or moderately (↓) according to [22].

**Table 8:** *KCNE1* VUSs reclassified as LP variants.

VUS	Cs	Functional Study ^a^	ClinPred	Paralogue
S^28^L	0.6	Smaller current	0.818	*KCNE5*-VUS:D^44^H
R^32^C	0.8	faster activation	0.622	*KCNE3*-VUS:R^47^G,*KCNE3*-VUS:R^47^Q, *KCNE3*-VUS:R^47^W
P^35^S	0.4	faster activation	0.957	*KCNE2*-VUS:V^41^A
L^48^I	0.9	~ current	0.963	*KCNE2*-Disease:M^54^T, *KCNE2*-VUS:M^54^V
L^48^F	0.9	faster activation	0.808	*KCNE2*-Disease:M^54^T, *KCNE2*-VUS:M^54^V
L^48^P	0.9		0.999	*KCNE2*-Disease:M^54^T, *KCNE2*-VUS:M^54^V
M^49^T	0.7		0.958	*KCNE5*-VUS:L^65^F
F^53^L	0.8	~ current	0.756	*KCNE2*-VUS:M^59^I, *KCNE5*-VUS:F^69^V
F^53^C	0.8	Small current	0.996	*KCNE2*-VUS:M^59^I,*KCNE5*-VUS:F^69^V
G^55^R	0.8		0.994	*KCNE2*-VUS:S^61^P
T^58^P	0.6	Slower activation	0.982	NA-Disease:T^58^P,*KCNE3*-VUS:V^72^G
T^58^A	0.6		0.987	NA-Disease:T^58^P,*KCNE3*-VUS:V^72^G
L^59^P	0.7	LoF	0.978	*KCNE2*-VUS:V^65^L,*KCNE2*-VUS:V^65^M, *KCNE5*-VUS:G^75^R
G^60^D	0.8	Small current	0.994	*KCNE2*-VUS:A^66^V,*KCNE3*-VUS:S^74^R, *KCNE5*-VUS:G^76^D
G^60^V	0.8		0.995	*KCNE2*-VUS:A^66^V,*KCNE3*-VUS:S^74^R, *KCNE5*-VUS:G^76^D
I^61^F	1		0.983	*KCNE2*-VUS:I^67^M
I^66^L	0.7		0.905	*KCNE3*-VUS:T^80^I
R^67^G	0.9	Small current	0.996	*KCNE3*-VUS:R^81^C
R^67^S	0.9	Small current	0.987	*KCNE3*-VUS:R^81^C
R^67^L	0.9	Small current	0.99	*KCNE3*-VUS:R^81^C
R^67^H	0.9	Small current	0.873	*KCNE3*-VUS:R^81^C
K^69^E	0.9		0.953	*KCNE3*-VUS:R^83^C,*KCNE3*-VUS:R^83^P, *KCNE5*-VUS:R^85^H
K^70^Q	0.9		0.977	*KCNE3*-VUS:K^84^E,*KCNE5*-VUS:K^86^E
K^70^M	0.9	Small current	0.994	*KCNE3*-VUS:K^84^E,*KCNE5*-VUS:K^86^E
K^70^E	0.9		0.983	*KCNE3*-VUS:K^84^E,*KCNE5*-VUS:K^86^E
L^71^V	0.4		0.956	*KCNE2*-Disease:R^77^W, *KCNE3*-VUS:V^85^A
E^72^K	0.4		0.984	*KCNE5*-VUS:V^88^D,^KCNE5^-VUS:V^88^I
H^73^R	0.6		0.931	*KCNE2*-VUS:H^79^R,*KCNE5*-VUS:E^89^K
S^74^W	0.4	Small current	0.998	*KCNE2*-VUS:S^80^P,*KCNE3*-VUS:R^88^C, *KCNE3*-VUS:R^88^H
S^74^P	0.4	Smaller current	0.629	*KCNE2*-VUS:^S80^P,*KCNE3*-VUS:R^88^C, *KCNE3*-VUS:R^88^H
Y^81^C	0.7	Smaller current	0.998	*KCNE2*-VUS:Y^87^C
I^82^M	0.4	Smaller current	0.885	*KCNE3*-VUS:I^96^S
I^82^V	0.4	Smaller current	0.978	*KCNE3*-VUS:I^96^S
I^82^F	0.4		0.993	*KCNE3*-VUS:I^96^S

^a^ ref. [41]; ~ current means “approximately the same current as in the wildtype channel”.

**Table 9:** AF3 model: *KCNE1* variants with WTRs, which contact WTRs of ClinVar-reported Kv7.1 variants.

*KCNE1* Variant	Classifi-cation	Current	Contact with			
		Change ^a^	*KCNE1*		Kv7.1	
M^1^L/I/R/T/K/V	P/VUS		R^67^G/S/L/H	VUS	F^256/5.529^	
L^48^I/F/P	LD VUS	~			I^138/1.555^ L^142/1.559^	
M^49^T/I	LD VUS		L^13^R/P/V/M	NP	C^331/6.545^Y T^327/6.541^D	LD VUS VUS
G^52^R/V/E/A	P/NP	R ↓			L^134/1.551^P	CIP
F^53^L/C	LD VUS	C ↓ L ~	L^13^R/P/V/M V^95^I	NP VUS	F^335/6.549^C F^270/5.543^	LD VUS
G^55^R	LD VUS				L^134/1.551^P F^130/1.547^	CIP
T^58^P/A	LD VUS	P ↓			F^130/1.547^ Y^267/5.540^F V^241/4.560^I	LD VUS LD VUS
L^59^P	LD VUS				F^127/1.544^L F^123/1.540^	NP
G^60^D/V	LD VUS	D ↓			I^263/5.536^V	VUS
I^61^F	LD VUS				Y^267/5.540^F D^242/4.561^Y/N/E Q^260/5.533^H I^263/5.536^V T^247/5.520^	LD VUS P/LP LD VUS VUS
I^66^L	LD VUS				F^123/1.540^ P^117/1.534^T/S/L	LP
R^67^G/S/L/H	LD VUS	All ↓	M^1^L/I/R/T/K/V	P/NP		

^a^ Measured for the indicated variant [41]; "~" means a similar current as in the wildtype channel; "↓" means the decreased current.

## Data Availability

The original contributions presented in this study are included in the article/Supplementary Materials. Further inquiries can be directed to the corresponding author.

## Supplementary Materials

The following supporting information can be downloaded at https://www.mdpi.com/article/10.3390/ijms26146561/s1.

## Abbreviations

AF3	AlphaFold3
AUC	Area under the ROC curve
CIP	Conflicting Interpretation of Pathogenicity
LD	Likely damaging variant
LQTS	Long QT Syndrome
MC	Monte Carlo
NP	Germline classification of pathogenicity is Not Provided
P/LP	Pathogenic/Likely Pathogenic variant
PLIC	P-loop ion channel
TM	Transmembrane
ROC	Receiver Operating Characteristic
VSD	Voltage-sensing domain
VUS	Variant of unknown clinical significance
WTR	Wildtype residue

## References

[1] Wu X., Larsson H.P. (2020). Insights into Cardiac IKs (KCNQ1/KCNE1) Channels Regulation. Int. J. Mol. Sci..

[2] Kekenes-Huskey P.M., Burgess D.E., Sun B., Bartos D.C., Rozmus E.R., Anderson C.L., January C.T., Eckhardt L.L., Delisle B.P. (2022). Mutation-Specific Differences in Kv7.1 (KCNQ1) and Kv11.1 (KCNH2) Channel Dysfunction and Long QT Syndrome Phenotypes. Int. J. Mol. Sci..

[3] Abbott J.W. (2014). Biology of the KCNQ1 Potassium Channel. New J. Sci..

[4] Jespersen T., Grunnet M., Olesen S.P. (2005). The KCNQ1 potassium channel: From gene to physiological function. Physiology.

[5] Sanguinetti M.C., Seebohm G. (2021). Physiological Functions, Biophysical Properties, and Regulation of KCNQ1 (K(V)7.1) Potassium Channels. Adv. Exp. Med. Biol..

[6] Sun J., MacKinnon R. (2017). Cryo-EM Structure of a KCNQ1/CaM Complex Reveals Insights into Congenital Long QT Syndrome. Cell.

[7] Bains S., Giammarino L., Nimani S., Alerni N., Tester D.J., Kim C.S.J., Christoforou N., Louradour J., Horvath A., Beslac O. (2024). KCNQ1 suppression-replacement gene therapy in transgenic rabbits with type 1 long QT syndrome. Eur. Heart J..

[8] Rothenberg I., Piccini I., Wrobel E., Stallmeyer B., Muller J., Greber B., Strutz-Seebohm N., Schulze-Bahr E., Schmitt N., Seebohm G. (2016). Structural interplay of K(V)7.1 and KCNE1 is essential for normal repolarization and is compromised in short QT syndrome 2 (K(V)7.1-A287T). Hear. Case Rep..

[9] Bellocq C., van Ginneken A.C., Bezzina C.R., Alders M., Escande D., Mannens M.M., Baro I., Wilde A.A. (2004). Mutation in the KCNQ1 gene leading to the short QT-interval syndrome. Circulation.

[10] Chen Y.H., Xu S.J., Bendahhou S., Wang X.L., Wang Y., Xu W.Y., Jin H.W., Sun H., Su X.Y., Zhuang Q.N. (2003). KCNQ1 gain-of-function mutation in familial atrial fibrillation. Science.

[11] Hateley S., Lopez-Izquierdo A., Jou C.J., Cho S., Schraiber J.G., Song S., Maguire C.T., Torres N., Riedel M., Bowles N.E. (2021). The history and geographic distribution of a KCNQ1 atrial fibrillation risk allele. Nat. Commun..

[12] Campuzano O., Sarquella-Brugada G., Brugada R., Brugada J. (2015). Genetics of channelopathies associated with sudden cardiac death. Glob. Cardiol. Sci. Pract..

[13] Albert C.M., MacRae C.A., Chasman D.I., VanDenburgh M., Buring J.E., Manson J.E., Cook N.R., Newton-Cheh C. (2010). Common variants in cardiac ion channel genes are associated with sudden cardiac death. Circ. Arrhythmia Electrophysiol..

[14] Kiper A.K., Wegner S., Kadala A., Rinne S., Schutte S., Winter Z., Bertoune M.A.R., Touska F., Matschke V., Wrobel E. (2024). KCNQ1 is an essential mediator of the sex-dependent perception of moderate cold temperatures. Proc. Natl. Acad. Sci. USA.

[15] Sachyani D., Dvir M., Strulovich R., Tria G., Tobelaim W., Peretz A., Pongs O., Svergun D., Attali B., Hirsch J.A. (2014). Structural basis of a Kv7.1 potassium channel gating module: Studies of the intracellular c-terminal domain in complex with calmodulin. Structure.

[16] Wrobel E., Tapken D., Seebohm G. (2012). The KCNE Tango—How KCNE1 Interacts with Kv7.1. Front. Pharmacol..

[17] Banyasz T., Jian Z., Horvath B., Khabbaz S., Izu L.T., Chen-Izu Y. (2014). Beta-adrenergic stimulation reverses the I Kr-I Ks dominant pattern during cardiac action potential. Pflug. Arch..

[18] Burg S., Attali B. (2021). Targeting of Potassium Channels in Cardiac Arrhythmias. Trends Pharmacol. Sci..

[19] Landrum M.J., Lee J.M., Benson M., Brown G.R., Chao C., Chitipiralla S., Gu B., Hart J., Hoffman D., Jang W. (2018). ClinVar: Improving access to variant interpretations and supporting evidence. Nucleic Acids. Res..

[20] Huang H., Kuenze G., Smith J.A., Taylor K.C., Duran A.M., Hadziselimovic A., Meiler J., Vanoye C.G., George A.L., Sanders C.R. (2018). Mechanisms of KCNQ1 channel dysfunction in long QT syndrome involving voltage sensor domain mutations. Sci. Adv..

[21] Phul S., Kuenze G., Vanoye C.G., Sanders C.R., George A.L., Meiler J. (2022). Predicting the functional impact of KCNQ1 variants with artificial neural networks. PLoS Comput. Biol..

[22] Vanoye C.G., Desai R.R., Fabre K.L., Gallagher S.L., Potet F., DeKeyser J.M., Macaya D., Meiler J., Sanders C.R., George A.L. (2018). High-Throughput Functional Evaluation of KCNQ1 Decrypts Variants of Unknown Significance. Circ. Genom. Precis. Med..

[23] Brewer K.R., Vanoye C.G., Huang H., Clowes Moster K.R., Desai R.R., Hayes J.B., Burnette D.T., George A.L., Sanders C.R. (2025). Integrative analysis of KCNQ1 variants reveals molecular mechanisms of type 1 long QT syndrome pathogenesis. Proc. Natl. Acad. Sci. USA.

[24] Richards S., Aziz N., Bale S., Bick D., Das S., Gastier-Foster J., Grody W.W., Hegde M., Lyon E., Spector E. (2015). Standards and guidelines for the interpretation of sequence variants: A joint consensus recommendation of the American College of Medical Genetics and Genomics and the Association for Molecular Pathology. Genet. Med..

[25] Dong C., Wei P., Jian X., Gibbs R., Boerwinkle E., Wang K., Liu X. (2015). Comparison and integration of deleteriousness prediction methods for nonsynonymous SNVs in whole exome sequencing studies. Hum. Mol. Genet..

[26] Niroula A., Vihinen M. (2016). Variation Interpretation Predictors: Principles, Types, Performance, and Choice. Hum. Mutat..

[27] Ghosh R., Oak N., Plon S.E. (2017). Evaluation of in silico algorithms for use with ACMG/AMP clinical variant interpretation guidelines. Genome Biol..

[28] Tordai H., Torres O., Csepi M., Padanyi R., Lukacs G.L., Hegedus T. (2024). Analysis of AlphaMissense data in different protein groups and structural context. Sci. Data.

[29] Anderson D., Lassmann T. (2018). A phenotype centric benchmark of variant prioritisation tools. NPJ Genom. Med..

[30] Tarnovskaya S.I., Korkosh V.S., Zhorov B.S., Frishman D. (2020). Predicting novel disease mutations in the cardiac sodium channel. Biochem. Biophys. Res. Commun..

[31] Walsh R., Peters N.S., Cook S.A., Ware J.S. (2014). Paralogue annotation identifies novel pathogenic variants in patients with Brugada syndrome and catecholaminergic polymorphic ventricular tachycardia. J. Med. Genet..

[32] Tarnovskaya S.I., Kostareva A.A., Zhorov B.S. (2021). L-Type Calcium Channel: Predicting Pathogenic/Likely Pathogenic Status for Variants of Uncertain Clinical Significance. Membranes.

[33] Tarnovskaya S.I., Kostareva A.A., Zhorov B.S. (2023). In silico analysis of TRPM4 variants of unknown clinical significance. PLoS ONE.

[34] Tikhonov D.B., Korkosh V.S., Zhorov B.S. (2025). 3D-aligned tetrameric ion channels with universal residue labels for comparative structural analysis. Biophys. J..

[35] Gigolaev A.M., Iurevaa D.A., Lagosha S.V., Brazhe A.R., Zhorov B.S., Vassilevski A.A. (2025). Golden Gate cloning enables efficient concatemer construction for biophysical analysis of heterozygous potassium channel variants from patients with epilepsy. Int. J. Biol. Macromol..

[36] Mandala V.S., MacKinnon R. (2023). The membrane electric field regulates the PIP(2)-binding site to gate the KCNQ1 channel. Proc. Natl. Acad. Sci. USA.

[37] Barro-Soria R., Perez M.E., Larsson H.P. (2015). KCNE3 acts by promoting voltage sensor activation in KCNQ1. Proc. Natl. Acad. Sci. USA.

[38] Kasuya G., Nakajo K. (2022). Optimized tight binding between the S1 segment and KCNE3 is required for the constitutively open nature of the KCNQ1-KCNE3 channel complex. eLife.

[39] Korkosh V.S., Zaytseva A.K., Kostareva A.A., Zhorov B.S. (2021). Intersegment Contacts of Potentially Damaging Variants of Cardiac Sodium Channel. Front. Pharmacol..

[40] Sun J., MacKinnon R. (2020). Structural Basis of Human KCNQ1 Modulation and Gating. Cell.

[41] Vanoye C.G., Desai R.R., John J.D., Hoffman S.C., Fink N., Zhang Y., Venkatesh O.G., Roe J., Adusumilli S., Jairam N.P. (2025). Functional profiling of KCNE1 variants informs population carrier frequency of Jervell and Lange-Nielsen syndrome type 2. bioRxiv.

[42] Jumper J., Evans R., Pritzel A., Green T., Figurnov M., Ronneberger O., Tunyasuvunakool K., Bates R., Zidek A., Potapenko A. (2021). Highly accurate protein structure prediction with AlphaFold. Nature.

[43] Evans R., O’Neill M., Pritzel A., Antropova N., Senior A., Green T., Green T., Bates R., Blackwell S., Yim J. (2021). Protein complex prediction with AlphaFold-Multimer. bioRxiv.

[44] Abbott G.W. (2016). KCNE1 and KCNE3: The yin and yang of voltage-gated K(+) channel regulation. Gene.

[45] Lundquist A.L., Manderfield L.J., Vanoye C.G., Rogers C.S., Donahue B.S., Chang P.A., Drinkwater D.C., Murray K.T., George A.L. (2005). Expression of multiple KCNE genes in human heart may enable variable modulation of I(Ks). J. Mol. Cell. Cardiol..

[46] UniProt C. (2015). UniProt: A hub for protein information. Nucleic Acids. Res..

[47] Dyer S.C., Austine-Orimoloye O., Azov A.G., Barba M., Barnes I., Barrera-Enriquez V.P., Becker A., Bennett R., Beracochea M., Berry A. (2025). Ensembl 2025. Nucleic Acids. Res..

[48] Boutet E., Lieberherr D., Tognolli M., Schneider M., Bansal P., Bridge A.J., Poux S., Bougueleret L., Xenarios I. (2016). UniProtKB/Swiss-Prot, the Manually Annotated Section of the UniProt KnowledgeBase: How to Use the Entry View. Methods Mol. Biol..

[49] Landrum M.J., Lee J.M., Benson M., Brown G., Chao C., Chitipiralla S., Gu B., Hart J., Hoffman D., Hoover J. (2016). ClinVar: Public archive of interpretations of clinically relevant variants. Nucleic Acids. Res..

[50] Karczewski K.J., Francioli L.C., Tiao G., Cummings B.B., Alföldi J., Wang Q., Collins R.L., Laricchia K.M., Ganna A., Birnbaum D.P. (2020). The mutational constraint spectrum quantified from variation in 141,456 humans. Nature.

[51] Kaltman J.R., Evans F., Fu Y.-P. (2018). Re-evaluating pathogenicity of variants associated with the long QT syndrome. J. Cardiovasc. Electrophysiol..

[52] Walsh R., Thomson K.L., Ware J.S., Funke B.H., Woodley J., McGuire K.J., Mazzarotto F., Blair E., Seller A., Taylor J.C. (2017). Reassessment of Mendelian gene pathogenicity using 7,855 cardiomyopathy cases and 60,706 reference samples. Genet. Med..

[53] Haitin Y., Wiener R., Shaham D., Peretz A., Cohen E.B., Shamgar L., Pongs O., Hirsch J.A., Attali B. (2009). Intracellular domains interactions and gated motions of I(KS) potassium channel subunits. EMBO J..

[54] Sievers F., Wilm A., Dineen D., Gibson T.J., Karplus K., Li W., Lopez R., McWilliam H., Remmert M., Söding J. (2011). Fast, scalable generation of high-quality protein multiple sequence alignments using Clustal Omega. Mol. Syst. Biol..

[55] Livingstone C.D., Barton G.J. (1993). Protein sequence alignments: A strategy for the hierarchical analysis of residue conservation. Comput. Appl. Biosci. CABIOS.

[56] Zvelebil M.J., Barton G.J., Taylor W.R., Sternberg M.J. (1987). Prediction of protein secondary structure and active sites using the alignment of homologous sequences. J. Mol. Biol..

[57] Golicz A., Troshin P.V., Madeira F., Martin D.M.A., Procter J.B., Barton G.J. (2018). AACon: A Fast Amino Acid Conservation Calculation Service.

[58] Liu X., Li C., Mou C., Dong Y., Tu Y. (2020). dbNSFP v4: A comprehensive database of transcript-specific functional predictions and annotations for human nonsynonymous and splice-site SNVs. Genome Med..

[59] Weiner S.J., Kollman P.A., Nguyen D.T., Case D.A. (1986). An all atom force field for simulations of proteins and nucleic acids. J. Comput. Chem..

[60] Garden D.P., Zhorov B.S. (2010). Docking flexible ligands in proteins with a solvent exposure- and distance-dependent dielectric function. J. Comput. Aided Mol. Des..

[61] Li Z., Scheraga H.A. (1987). Monte Carlo-minimization approach to the multiple-minima problem in protein folding. Proc. Natl. Acad. Sci. USA.

[62] Zhorov B.S. (1981). Vector method for calculating derivatives of energy of atom-atom interactions of complex molecules according to generalized coordiantes. J. Struct. Chem..

[63] Long S.B., Tao X., Campbell E.B., MacKinnon R. (2007). Atomic structure of a voltage-dependent K+ channel in a lipid membrane-like environment. Nature.

[64] Zhorov B.S. (2021). Possible Mechanism of Ion Selectivity in Eukaryotic Voltage-Gated Sodium Channels. J. Phys. Chem. B.

