# Supplementary material for: Predicting the Damaging Potential of Uncharacterized KCNQ1 and KCNE1 Variants
Source: Int J Mol Sci. 2025 Jul 8;26(14):6561. doi: 10.3390/ijms26146561 (PMC12294243; doi:10.3390/ijms26146561)
Supplement: Supplementary file 1 [file ijms-26-06561-s001.zip › ijms-3705358_Supplementary_Figure S1.pdf]

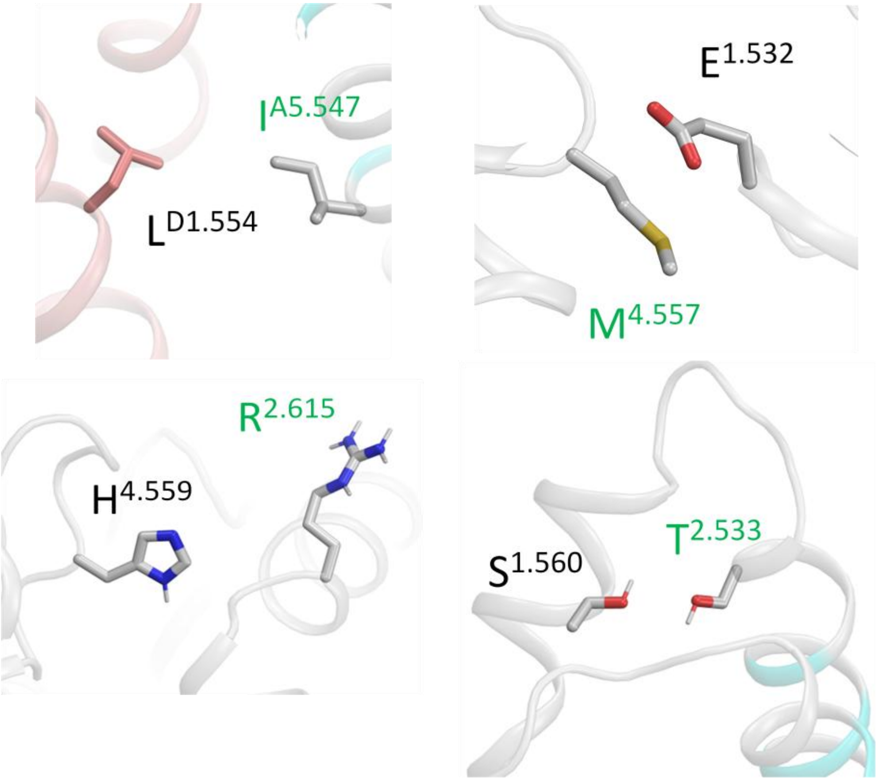

B/LB variant	LD variant
$I^{274/5.547}V$	$L^{137/D1.554}P$
$M^{238/4.557}V$	$E^{115/1.532}D$
$R^{195/2.615}Q$	$H^{240R/4.559}Q$
$T^{153/2.533}M$	$S^{143/1.560}F$

Supplementary Figure S1. WTRs of KCNQ1 benign/likely benign (B/LB) variants that form intersegmental contacts with WTRs of likely damaging (LD) variants. WTRs of B/LB variants are indicated by green labels.
